# Supplementary material for: Heart Rate in Patients with SARS-CoV-2 Infection: Prevalence of High Values at Discharge and Relationship with Disease Severity
Source: J Clin Med. 2021 Nov 28;10(23):5590. doi: 10.3390/jcm10235590 (PMC8658577; doi:10.3390/jcm10235590)
Supplement: Supplementary file 1 [file jcm-10-05590-s001.zip › jcm-1488334-supplementary.pdf]

Table S1. COVID-19 related treatments and their association with heart rate at discharge in hospitalized patients with SARS-CoV-2 infection.

	Models Adjusted only by Heart Rate at Admission		Fully Adjusted Model *	
	**Coefficient (95% CI)**	***p***	**Coefficient (95% CI)**	***p***
Tocilizumab vs no	2.31 (−0.77;5.39)	0.142	2.15 (−0.90;5.20)	0.166
Azithromycin vs no	−0.40 (−2.63;1.83)	0.727	−0.26 (−2.57;2.06)	0.826
Hydroxychloroquine-chloroquine vs no	−2.09 (−4.18;0.01)	0.051	−2.29 (−4.77;0.19)	0.070
Corticosteroids vs no	0.17 (−0.84;3.02)	0.268	0.97 (−0.97;2.92)	0.326

* Intercept (std. error) = 98.860 (24.840), adjusted R^2 = 13.6%; adjusted by: Heart rate at admission, age, gender, Charlson Comorbidity Index, oxygen saturation, systolic blood pressure, body-temperature, hemoglobin, need for intensive care, COVID-19 pulmonary disease, drugs acting on heart rate.

Table S2. Logistic regression showing the association of predictors with sinus tachycardia (heart rate at discharge > 100 bpm) in hospitalized patients with SARS-CoV-2 infection.

	**OR (95% CI)**	**P**
Heart rate at admission, per bpm increase	1.016 (0.995;1.038)	<0.001
Age, per year increase	0.972 (0.936;1.009)	0.511
Gender, female as reference	1.549 (0.64;3.754)	0.935
Charlson Comorbidity Index, per unit increase	1.073 (0.856;1.345)	0.195
Oxygen saturation, %, per unit increase	0.961 (0.885;1.044)	0.506
Systolic blood pressure, per 1 mmHg increase	0.996 (0.979;1.014)	0.640
Body-temperature ° Celsius, per unit increase	1.182 (0.805;1.736)	0.348
Hemoglobin, per g/dL increase	0.771 (0.619;0.961)	0.023
Severe disease, absence as reference	3.783 (1.539;9.299)	<0.001
COVID-19 pulmonary disease, absence as reference	1.263 (0.336;4.746)	0.348
Drugs acting on heart rate, absence as reference	0.547 (0.205;1.457)	0.097

COVID, COronaVIrus-related Disease; OR, Odds Ratio;
